# Zooming into plant-flower visitor networks: an individual trait-based approach

**DOI:** 10.7717/peerj.5618

**Published:** 2018-09-17

**Authors:** Beatriz Rumeu, Danny J. Sheath, Joseph E. Hawes, Thomas C. Ings

**Affiliations:** 1 Applied Ecology Research Group, Department of Biology, Anglia Ruskin University, Cambridge, United Kingdom; 2 Terrestrial Ecology Group, Mediterranean Institute of Advanced Studies (CSIC-UIB), Mallorca, Balearic Islands, Spain; 3 Institute of Global Health, Faculty of Medicine, University of Geneva, Geneva, Switzerland; 4 School of Biological and Chemical Sciences, Queen Mary University of London, London, United Kingdom

**Keywords:** Bee–flower interactions, Cluster analysis, Intertegular distance, Nectar holder depth, Proboscis length, Pollination

## Abstract

Understanding how ecological communities are structured is a major goal in ecology. Ecological networks representing interaction patterns among species have become a powerful tool to capture the mechanisms underlying plant-animal assemblages. However, these networks largely do not account for inter-individual variability and thus may be limiting our development of a clear mechanistic understanding of community structure. In this study, we develop a new individual-trait based approach to examine the importance of individual plant and pollinator functional size traits (pollinator thorax width and plant nectar holder depth) in mutualistic networks. We performed hierarchical cluster analyses to group interacting individuals into classes, according to their similarity in functional size. We then compared the structure of bee-flower networks where nodes represented either species identity or trait sets. The individual trait-based network was almost twice as nested as its species-based equivalent and it had a more symmetric linkage pattern resulting from of a high degree of size-matching. In conclusion, we show that by constructing individual trait-based networks we can reveal important patterns otherwise difficult to observe in species-based networks and thus improve our understanding of community structure. We therefore recommend using both trait-based and species-based approaches together to develop a clearer understanding of the properties of ecological networks.

## Introduction

During recent decades, ecological networks have become an increasingly useful tool to capture the mechanisms underlying plant-animal assemblages (Bascompte & Jordano, 2007; Reiss et al., 2009; Heleno et al., 2014; Ings & Hawes, 2018). The vast majority of mutualistic networks published to date are composed of nodes representing plant and animal species that are connected by edges indicating the presence of interactions between them. This approach provides a holistic picture of the community structure, and allows the detection of patterns that cannot be inferred from the observations of the nodes (species) in isolation (Proulx, Promislow & Phillips, 2005; Bascompte & Jordano, 2014). However, in such species-based networks, nodes consist of populations of conspecific individuals that may vary in many biological traits such as phenotype, phenology or behaviour. For instance, different individuals of a bee species vary in sex, age class, body size and behaviour, which can positively or negatively affect their efficiency in pollinating certain flowers (Bolnick et al., 2003; Araújo, Bolnick & Layman, 2011). Therefore, there is growing recognition that potentially important information is lost when averaging species data and ignoring inter-individual variation (Ings et al., 2009; Olesen et al., 2010; Tur et al., 2014; Kuppler et al., 2016; Ings & Hawes, 2018).

Several recent studies on pollination interactions have stressed the importance of scaling down from species-based plant-flower visitor networks to individual-based networks (Gómez, Perfectti & Jordano, 2011; Dupont, Trøjelsgaard & Olesen, 2011; Gómez & Perfectti, 2012; Dáttilo et al., 2014; Dupont et al., 2014; Tur et al., 2014; Tur, Olesen & Traveset, 2015; Kuppler et al., 2016; Valverde, Gómez & Perfectti, 2016). These studies have revealed how inter-individual variation generates link patterns that may have important implications for community dynamics and species stability. Conspecific individuals within a population (both plants and pollinators) have been shown to vary widely in the number of interacting partners (Gómez, Perfectti & Jordano, 2011; Dupont, Trøjelsgaard & Olesen, 2011), degree of specialization (Tur et al., 2014), spatial patterns (Dupont et al., 2014) or even temporal schedules (Valverde, Gómez & Perfectti, 2016). However, individual-based studies of plant-flower visitor networks are still scarce (Kuppler et al., 2016; Kuppler et al., 2017), and they have only focused on the intraspecific variation of one or two species in which different individuals have been represented as nodes (Dupont, Trøjelsgaard & Olesen, 2011; Dupont et al., 2014; Kuppler et al., 2016). Covering a more diverse community therefore represents the next step to extend our understanding of plant-flower visitor interaction networks.

Traits play an important role for ecosystem functioning through mechanisms of resource use complementarity and identity effects, and intraspecific trait variation can alter ecological dynamics by multiple mechanisms (Bolnick et al., 2011; González-Varo & Traveset, 2016). A trait-based approach, zooming in on individual phenotypic variation (Woodward et al., 2005; Woodward et al., 2010b; Woodward & Warren, 2007) in contrast to the broad traditional species focus, allows us to move towards a more accurate mechanistic understanding of network structure (Woodward et al., 2010a). Body size is recognised as a major determinant of network structure, particularly in predator–prey food webs, with examples from aquatic systems displaying an increase in body size over orders of magnitude moving up the food chain (Woodward et al., 2010b; Gravel et al., 2013). Although there is significantly lower intraspecific size variation in plant-flower visitor networks than in aquatic food webs (Ings et al., 2009), the size traits of interacting partners (e.g., nectar holder depth in flowers and tongue length in visitors) are still of direct relevance for the occurrence of particular interactions (Harder, 1983; Peat, Tucker & Goulson, 2005; Stang, Klinkhamer & Van der Meijden, 2006a; Stang, Klinkhamer & Van der Meijden, 2006b; Stang et al., 2009).

We develop an individual trait-based approach to constructing flower-visitor networks based on functional size of individual bees and the flowers they visit. To introduce and test this new approach we use a single network of bee-plant interactions observed over two summers in a small English meadow. Because tracking individual bees and scoring traits for all the flowers they interact with is logistically challenging, we instead record the functional size traits of both bees and flowers recorded during single interactions. This novel individual-based network, using traits to assign nodes, is then compared to a traditional species-based network to assess the potential for this approach to increase our understanding of the mechanisms operating in mutualistic networks. If flower choice by bees (or the probability of a given interaction occurring) is driven by intraspecific trait variation more or less than by species identity, we would expect structure to differ between species-based and individual-based networks. The use of trait data at the resolution of individuals to assign network nodes provides an alternative or complementary framework to the traditional process that simplifies interactions by using species, or average trait values per species, as nodes.

## Material and Methods

### Study system

We conducted fieldwork in a 0.52 ha flower-rich meadow (UK National Vegetation Classification MG5–unimproved old hay meadow) located at the Roding Valley Nature Reserve (Essex, UK [51°38′04.1″N 0°04′12.6″E]) (Fig. S1). The surrounding landscape comprised similar meadows with associated hedgerows and trees. Due to the labour-intensive effort required to sample traits for the interacting partners of the entire flower visitor community, we focused on bees, which stand out among insects as the world’s primary pollinators in most ecosystems (Neff & Simpson, 1993; Winfree, 2010). Our study site supported ten species (four families) of flowering plants and 28 species (six families) of bee (Tables S1, S2).

### Sampling procedure

We conducted surveys over 5 days from 29 June to 5 July 2011, and 4 days from 13 June to 28th June 2012, using two observers to record interactions between bees and flowers along the transects. These short periods represent the peak period for the meadow which is cut for hay in July, thus removing all flowers. The transects were positioned to maximise coverage of all possible interactions in the meadow. In 2011 this resulted in an approximately ‘L’-shaped transect, with an angle of 105° between the two sections (Fig. S1A) and a total length of 135 m. To increase coverage of the meadow in 2012, we changed the transect into four separate sections (75, 53, 31, and 17 m) perpendicular to the length of the field with a total length of 176 m (Fig. S1B). Interactions were observed up to 1 m either side of the transects, meaning an effective coverage of 270 m^2^ (5.2% of the total meadow area) in 2011 and 352 m^2^ (6.8% of the total meadow) in 2012. To allow for diurnal turnover in interactions, the transects were walked twice per day in opposite directions between 10:00–13:00 and 12:00–16:00 in 2011. Due to increased duration of transect walks, and collection of other data, transects in 2012 were only walked once per day in opposite directions on consecutive days. Surveys lasted approximately 2 h per transect walk in 2011, with one transect conducted on the first day and two transects on all other days (approximately 15 h observations). In 2012, surveys lasted between 2 and 4 h. Total survey time across the two years was approximately 28 h. Surveys in both years were conducted during fine weather (the weather did not change during the course of any survey and no transects needed to be abandoned).

On each transect survey, one observer noted the type of interaction (nectar and/or pollen collection, or nectar robbing) for bees visiting flowers. Once the interaction was observed, bees were captured and the second observer measured the maximum floral display size of the flower being visited. We defined floral display size as the width at the widest point of a single flower, or in the case of Asteraceae and Apiaceae, a single inflorescence. We placed bees that could be identified in the field (e.g., *Bombus* spp.) in a bee-marking cage, positioned so that their bodies were flat and their tegulae were visible, and took digital photographs of them against a scale to allow the intertegular distance to be measured using image analysis software (ImageJ v1.46r). Prior to their release at the same location as they were captured, we marked all bees with a dot of non-toxic marker paint (Posca PC-5M, Uni, Japan) on the dorsal surface of the thorax to avoid any re-recording of the same individual. For any bees requiring laboratory identification, we used individual collection vials charged with ethyl acetate, and took a digital photograph of the pinned specimen to measure intertegular distance and proboscis length using the same image analysis software as above.

Both proboscis length and nectar holder depth are considered as pivotal functional traits in plant–pollinator interactions (Harder, 1983; Stang, Klinkhamer & Van der Meijden, 2006a; Stang, Klinkhamer & Van der Meijden, 2006b; Stang et al., 2009). Therefore, we used collected specimens of each bee species encountered (*n* = 1 to 29 depending upon encounter rate per species) to define the relationship between intertegular distance and proboscis length to enable us to use intertegular distance measured in the field to predict the proboscis length of bees from all observed interactions. We also collected a subsample of open flowers from each flowering plant species (5–10 flowers per plant, 1–5 plants per species; *n* = 5 to 25) to define the relationship between maximum floral display size and nectar holder depth per plant species. Regressions of nectar holder depth against maximum floral display size for each species were used to predict the nectar holder depth of flowers whose floral display size was measured during the transects. For species with nectar holder depths that were effectively too short to measure, we assigned a value of 1 mm for the nectar holder depth of all individuals (four out of 10 species). Due to the high abundance of certain bee species, such as honeybees (*Apis mellifera*) and bumblebees (*B. lapidarius*), and the time-consuming nature of trait measurements, individual measurements were only taken for the first 50 interactions observed in these cases. This allowed a wider range of unique individual interactions to be characterised.

### Construction of flower-visitation networks

We used the frequency of flower visits by bees as the interaction weight to construct two different types of quantitative bipartite networks: (i) a traditional species-based network, and (ii) our novel individual trait-based network. For the trait-based network, we used the functional size measurements obtained from individual plants and bees. Predicted nectar holder depth (NHD; Fig. S2A) was used as the functional size trait to characterize our focal flowering plant community. We selected intertegular distance (ITD) as our measure of functional size in bees as it was highly correlated with proboscis length (Fig. S2B) and it captures other variables such as flight range (Greenleaf et al., 2007). This high-resolution individual data on trait variation (NHD and ITD) was used to perform cluster analyses and objectively allocate all interacting individuals into more realistic trait groups, which were then treated as nodes in the subsequent networks.

All analyses were performed in R 3.2.4 (R Development Core Team, 2015). As both NHD and ITD are continuous variables, we computed an agglomerative hierarchical clustering with the function *agnes* available in the *cluster* package (Maechler et al., 2016), using Euclidean distances for calculating dissimilarities between individuals. Agglomerative clustering starts with each individual contributing to the cluster analysis being treated separately, and then joins individuals into clusters based on the distance metric. Firstly, in order to compare the traditional species-based network with our trait-based network more directly, we ran the analysis while setting the number of clusters in the trait-based network to match the number of plant and bee species (following Woodward et al., 2010b) and remove the effect of network size on network properties. We refer to this hereafter as ‘constrained’ functional size-based network. Secondly, we explored the optimal number of clusters in which individual bees and flowers of the community group according to their trait variation (and irrespective of species), and constructed the resulting ‘unconstrained’ functional size-based network. To compute these cluster analyses, we used the *NbClust* package (Charrad et al., 2014), which provides up to 30 indices for determining the optimal number of clusters in the dataset. This approach offers the best clustering scheme by varying all combinations of number of clusters, distance measures, and aggregation methods. We selected the “average” aggregation method, which uses the average pairwise distance between all pairs of individuals in the different clusters as the measure of distance (Sokal & Michener, 1958). Network figures were drawn using the *bipartite* package (Dormann, Gruber & Fründ, 2008).

### Network parameters and data analysis

We used four commonly used quantitative network parameters (weighted connectance, weighted nestedness, interaction evenness and degree of complementary specialization) and two node-level parameters (specialization and strength) to describe and compare the structure of the networks constructed:

*Weighted connectance* (*C*_*q*_) (e.g., Kaiser-Bunbury et al., 2011): gives individual weight to each node based on their total interaction frequency, in contrast to qualitative connectance (the fraction of all possible links that are realized in a network), and, therefore, better captures the functional importance of a species (or functional group) in the community.

*Weighted nestedness (WNODF)* (Almeida-Neto & Ulrich, 2011): uses quantitative data to give a measure of the degree of hierarchy in the organization of the interactions. In our case this relates to size structuring of functional size based network. In a nested network, nodes with fewer interactions (specialists) are mostly linked with a subset of nodes linked to the most connected ones (generalists). Nestedness ranges from zero (not nested) to 100 (highly nested).

*Interaction evenness* (*IE*) (Tylianakis, Tscharntke & Lewis, 2007): is based on Shannon’s evenness and measures the uniformity of interactions among nodes in a network. IE ranges from zero (complete unevenness in the distribution of interaction frequencies) to one (complete uniformity).

*Degree of complementarity specialization* (*H*_2_′) (Blüthgen, Menzel & Blüthgen, 2006): is a measure of specialization that depicts how much the interactions of each node differ from each other in the network. *H*_2_′ ranges between zero (no specialization) and one (complete specialization).

*Specialization* (*d*′) (Blüthgen, Menzel & Blüthgen, 2006) for plants (*d*′_*p*_) and bees (*d*′_*b*_): gives levels of specialization of each species (or functional trait node in our case). It accounts for the available resources provided by the interaction partners, so a pollinator species (or group of species included in a node) that visits resources proportionally to the total number of interactions of the species (functional size nodes) it interacts with is considered generalized, while a species that visits rare resources disproportionately is considered specialized. The index ranges from zero (highly opportunistic) to one (highly selective).

*Strength* of a bee species (or node) (*st*′_*b*_) is a measure of the importance of a pollinator from the perspective of the flowering plant community (Bascompte, Jordano & Olesen, 2006). It is the sum of dependencies of the plants relying on that particular pollinator. In the same way, the strength of a plant species (or species included in a node) (*st*′_*p*_*)* is the sum of dependencies of the pollinators relying on that given plant species. In the case of the bees, for instance, it is calculated as the relative frequency of a bee species (or bee size cluster) on a particular plant species (or plant size cluster), i.e., the number of interactions between pollinator nodes *j* and plant nodes *i* divided by number of visits of all pollinator nodes to plant node *i*.

Network parameters were calculated using the *bipartite* package (Dormann et al., 2009) run in R 3.2.4. (R Development Core Team, 2015), testing their significance against 1,000 networks generated by the null model r2dtable (function ‘*nullmodel*’ in *bipartite*) based on the Patefield algorithm (Patefield, 1981), and using a *z*-score test. We also tested the significance for nestedness in our networks using three different null models (CRT, Conserve Row Totals; CCT, Conserve Column Totals; and RCTA, Row Column Total Average), implemented in FALCON (Beckett, Boulton & Williams, 2014). We used the adaptive ensemble method to reduce undersampling of the null distribution and to optimize the minimal ensemble size sufficiently to give robust statistics.

Finally, we also computed centrality to evaluate the node’s relative importance to the structure of the network and identify key species and functional size ranges in the community (Martín González, Dalsgaard & Olesen, 2010; Gómez & Perfectti, 2012; Mello et al., 2015). Centrality indicates how well connected a node is to the rest of the nodes in the network; central nodes can trigger a rapid breakdown of the network structure if they are selectively removed (e.g., Memmott, Waser & Price, 2004). We estimated centrality using three metrics (see Mello et al., 2015 for details): (i) normalized degree centrality, i.e., the proportion of different partners a certain node interacts with in relation to the number of potential partners in the network, (ii) closeness centrality, i.e., the shortest path from one node to all other nodes in the network, and (iii) betweenness centrality, i.e., the importance of a node for connecting different parts of the network. Centrality measures were computed using the software Pajek 4.10 (Batagelj & Mrvar, 1998). To test whether the node-level parameters varied significantly between the species-based network and the constrained trait-based network, we performed overall generalized linear models (GLM) and linear regressions (according to the distribution of the metric values).

## Results

### Interacting partners and phenotypic traits

We recorded a total of 272 individual bee-flower interactions within our community of 10 plant and 28 bee species (Tables S1, S2). For plant traits, nectar holder depth (range = 0.98–11.63 mm) of sampled flowers (*n* = 131 flowers from the six out of 10 plant species with measurable nectar holder depths) was positively correlated (mean *R*^2^ = 0.516 ± 0.101) with maximum floral display size (range = 7.0–53.44 mm) for most (four out of six) of the plant species subsampled for trait measurement (Fig. S2A). For bee traits, intertegular distance (range = 1.28–5.47 mm) was positively correlated (*R*^2^ = 0.817) with proboscis length (range = 1.19–7.86 mm) across the subset of individuals (this included those collected during this experiment and some additional bees from a private collection held by T. Ings) measured in the laboratory (Fig. S2B).

### Species-based network versus constrained functional trait-based network

**Figure 1:**
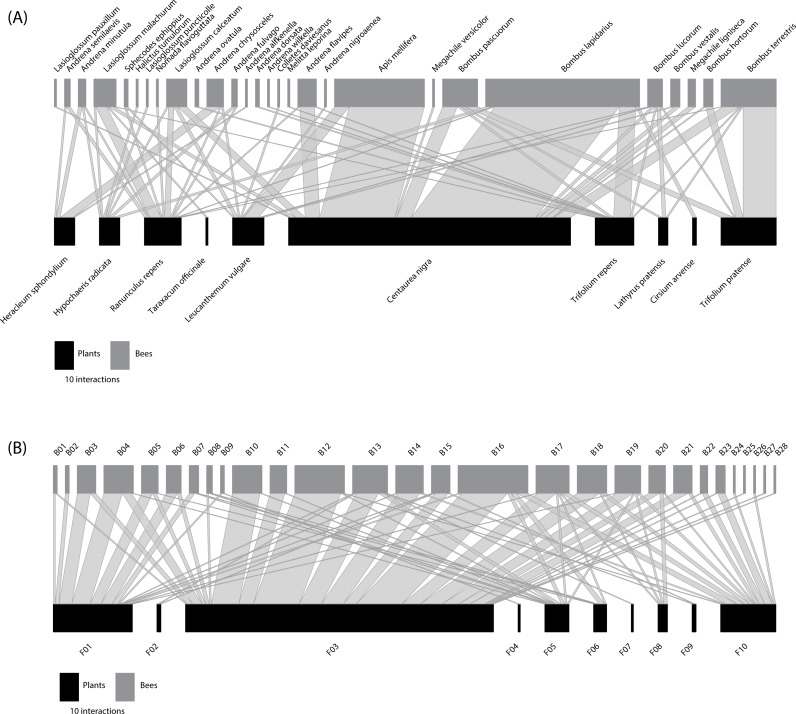
Comparison of traditional species-based and individual trait-based approaches to constructing plant-flower visitor networks. (A) Species-based network; (B) *Constrained* (the number of bee and plant nodes set the same as the species-based network) functional size-based network. Bee (grey rectangles) and plant (black rectangles) nodes are shown in the upper and lower levels respectively. Nodes are sorted from left to right, from smallest to largest size (see Tables S1–S4 for details). The two networks are quantitative, i.e., the length of the rectangles are proportional to the number of interactions of each node and the width of the edges indicates the interaction frequency between nodes.

The species-based network (Fig. 1A) comprised 10 plant species from four families (Table S1) and 28 bee species from six families (Table S2). Predicted NHD of flowers varied from 1.00 (open flowers) to 10.00 mm, whereas the intertegular distance of bees varied from 1.37 to 6.42 mm. In the nodes of the equivalent functional size-based network, where the number of nodes was constrained to match that of the species-based network (Fig. 1B), the number of species grouped within nodes ranged from 1 to 4 species in the case of the flowers visited (Table S3), and from 1 to 9 species in the case of the bees (Table S4). See also Fig. S3 for the distribution of the interacting flowers and bees in their respective size-based clusters. The interaction matrices (Fig. S4) and network representations (Fig. 1) of the two networks depict a regular pattern of flower-bee interactions that reveals the important role of body size. This pattern is more evident in the functional size-based network, where all intraspecific variation in body size has been considered, and size ranges do not overlap among nodes. In this network, a general pattern of size matching can be observed between the NHD of the flowers and the ITD of the bees (see Fig. 1B, where nodes have been ordered by size).

The two networks were quite similar in their structural parameters, although the species-based network showed lower values in *C*_*q*_, *IE* and *WNODF* (Table 1 and Table S5). However, the species-based network was more specialized (*H*_2_′) compared to its functional size-based counterpart (Table 1). By changing from the taxonomic to the functional trait-based representations, nestedness was the most affected parameter, jumping from 17.38 in the species-based network to 33.32 (Table S5) in the functional size-based network. The two webs showed significantly lower values of connectance (*C*_*q*_) and interaction evenness (*IE*) than the randomly assembled networks (Table 1). However, both networks were significantly more specialised (*H*_2_′) than the networks generated by the null model (Table 1). At the node level, bees were significantly more specialized in the species-based network compared to the functional size-based network (*F*_1,54_ = 13.43, *P* < 0.001). There was no significant variation in the specialization degree of plants between the networks, and strength did not vary among networks for either bees or plants (Table 1).

**Table 1:** Network parameters for the species-based, constrained and unconstrained functional size-based networks.

Parameters	Species-based network	Constrained size-based network	Unconstrained size-based network
*P*	10	10	5
*A*	28	28	4
*C*_*q*_	0.100 (*z* = 13.20^***^)	0.195 (*z* = 8.69^***^)	0.282 (*z* = 27.74^***^)
*IE*	0.558 (*z* = 20.54^***^)	0.661 (*z* = 10.00^***^)	0.699 (*z* = 28.62^***^)
*H*_2_′	0.460 (*z* = − 20.54^***^)	0.332 (*z* = − 9.97^***^)	0.353 (*z* = − 28.62^***^)
*WNODF*	17.382 (*z* = 5.43)	33.324 (*z* = 3.10)	51.042 (*z* = − 1.00)
*d*′_*b*_ (X ± SD)	0.326 ± 0.179	0.170 ± 0.140	0.232 ± 0.154
*d*′_*p*_ (X ± SD)	0.447 ± 0.208	0.331 ± 0.204	0.224 ± 0.182
*str*′_*b*_(X ± SD)	0.357 ± 0.518	0.357 ± 0.490	1.250 ± 0.859
*str*′_*p*_(X ± SD)	2.800 ± 2.424	2.800 ± 4.527	0.800 ± 0.893

**Notes.**

*P* number of flower-nodes *A* number of bee-nodes *C*_*q*_ weighted connectance *IE* interaction evenness *H*_2_′ network specialization index *WNODF* weighted nestedness *d*′_*b*_ and *d*′_*p*_ species specialization index for bees and plants, respectively *str*′_*b*_ and *str*′_*p*_ strength for bees and plants, respectively

For the network-level parameters, asterisks indicate the probability that the observed values differ significantly from mean values obtained from null models: *, *P* < 0.05; **, *P* < 0.01; and ***, *P* < 0.001.

Positive *z*-values indicate that the observed value is lower than the mean value of the null model. See Table S5 for details on *WNODF* values.

Centrality varied between the two networks, as shown by the linear model fitted for the closeness metric in the case of bee-nodes (*F*_1,54_ = 22.39, *P* <0.001). The mean value of closeness in the species-based network (0.36 ± 0.06) was significantly lower than that of the functional trait-based network (0.42 ± 0.07). While *B. lucorum* stands out for its central position in the species-based network (Table S6 : normalized degree = 0.60, closeness = 0.47, betweenness = 0.14), the node with highest value of centrality was B13 (normalized degree = 0.60, closeness = 0.49, betweenness = 0.12) for the size-based network. B13 groups individuals of three different species of Apidae (*A. mellifera*, *B. lapidarius* and *B. pascuorum*) with intertegular distances ranging from 3.69–3.83 mm (Table S4). For the plants, *Centaurea nigra* showed the most central position (normalized degree = 0.46, closeness = 0.46, betweenness = 0.31) in the species-based network, whereas the node F03 was the most central in the functional trait-based network (normalized degree = 0.82, closeness = 0.64, betweenness = 0.52). This flower node (F03), with a NHD ranging from 3.21 to 4.15 mm (Table S3), comprised flowers of two plant species: *C. nigra* and *Leucanthemum vulgare*.

### Unconstrained functional trait-based network

**Figure 2:**
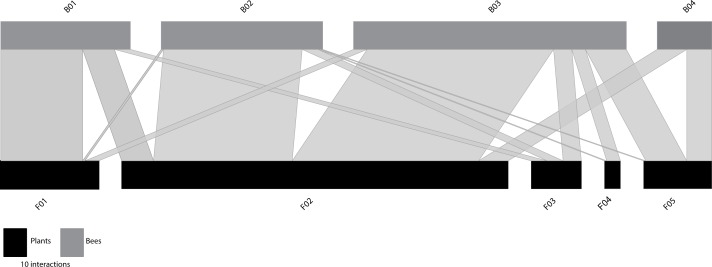
*Unconstrained* functional size-based network built independently of the number of interacting species. Bee (grey rectangles) and plant (black rectangles) nodes are shown in the upper and lower levels respectively. Nodes are sorted from left to right, from smallest to largest size (see Tables S7–S8 for details). The length of the rectangles are proportional to the number of interactions of each node and the width of the edges indicates the interaction frequency between nodes.

When the functional size-based network was constructed with the optimal number of clusters according to trait variation (Fig. 2), it comprised five flower nodes that encompassed one to four plant species (Table S7), and four bee nodes that covered seven to 17 species (Table S8). Figure S5 shows the distribution of the interacting flowers and bees in their respective size-based clusters. A general pattern of size matching between bees and flowers was also evident in this unconstrained functional size-based network (Fig. 2). Thus, bees with longer proboscises tended to interact more frequently with flowers with deeper nectar holder tubes, whereas bees with shorter proboscises tended to interact more frequently with flowers with shallow nectar holder tubes.

Like the constrained functional size-based networks (with equal number of nodes as interacting species), the unconstrained network was significantly more specialized than the randomly assembled networks of the null models (Table 1). It also had significantly lower values of *C*_*q*_ and *IE* than the randomly assembled networks, a pattern also observed for the constrained network. The observed value of nestedness did not differ significantly from the null models (Table 1, Table S5).

At the node level, the centrality metrics (see Table S9) show that among the plants the node F02, composed of *C. nigra* and *L. vulgare,* with a NHD ranging from 3.02 to 4.15 mm (Table S7), occupied the most central position. Among bees, those with highest values of centrality were B02 and B03 (both with normalized degree = 1.00, closeness = 0.73, betweenness = 0.23). These nodes include bees belonging to four different families that show intermediate sizes (Andrenidae, Apidae, Megachilidae and Melittidae), and cover intertegular distances ranging from 3.20 to 3.83 mm and from 3.88 to 5.05, respectively (Table S8).

## Discussion

To date, the taxonomic grouping of interacting individuals has dominated the way in which we represent and interpret complex communities. However, this approach does not take into account the importance of intraspecific variability and thus, could be limiting our understanding on the drivers determining the assemblage of interacting partners (Woodward et al., 2010b; Bolnick et al., 2011; González-Varo & Traveset, 2016; Kuppler et al., 2016; Kuppler et al., 2017). Our study shows that the incorporation of individual functional trait variability in mutualistic networks is a suitable analytical framework. While we cannot make strong conclusions based on our analysis of a small, but unique network, we have demonstrated that our approach has the potential to reveal important patterns that could be masked when using only a taxonomic-based approach. We found that by using functional size traits as the level of organization, a single node of our networks grouped bees from up to four different families and 17 species (three families and nine species when nodes were established while constraining the size of the network according to the number of interacting species). This reflects the great differences that our perception of how to group individuals (taxonomic versus trait-based) can create when building mutualistic networks (Raffaelli, 2007).

When contrasting the species-based network with the constrained functional size-based network, a general pattern of size matching is evident. Recent work has shown a strong correlation between intertegular distance and proboscis length in bees (Cariveau et al., 2016), which is supported by our study (Fig. S2B). In the trait-based networks we found that larger bees (with longer proboscises), irrespective of species, interacted more frequently with flowers that had deeper nectar holder tubes, whereas smaller bees (with shorter proboscises) interacted more frequently with flowers that had more shallow nectar holder tubes. This pattern indicates that body size plays an important role in structuring the general linkage pattern of the community. While this has been previously reported in food webs (Woodward et al., 2005; Woodward et al., 2010b; Gravel et al., 2013), where predators are gape limited, we have shown that similar patterns can be expected in mutualistic systems too, where size traits are also highly relevant to function (Harder, 1983; Peat, Tucker & Goulson, 2005; Stang, Klinkhamer & Van der Meijden, 2006a; Stang, Klinkhamer & Van der Meijden, 2006b; Stang et al., 2009). For example, intertegular distance acts as a proxy of proboscis length, which is a phenotypic trait of paramount importance in multiple functions of bee ecology and evolution, including flower choice (Peat, Tucker & Goulson, 2005; Goulson, Lye & Darvill, 2008). Intertegular distance is also a good predictor of foraging distance (Greenleaf et al., 2007) and, as a measure of body size, it is related to energy requirements (Osorio-Canadas et al., 2016).

The size-matching arrangement observed in the functional size-based network resulted in a more symmetric linkage pattern, i.e., greater interaction evenness and weighted connectance, than observed for the species-based network. Kuppler et al. (2016), who compared individual plant-flower visitor networks with traditional plant species-flower visitor species networks also found greater interaction evenness and connectance when individual interactions were considered. Recent work has demonstrated an important relationship between weighted connectance and the skewness of the distributions of fluxes and interaction strengths in food webs (Van Altena, Hemerik & De Ruiter, 2016), showing a positive correlation between weighted connectance and stability. Therefore, while we need to interpret these results with caution due to the small size of our network, the differences in structural properties between species and trait-based networks (also shown by Kuppler et al., 2016) could provide new insights into the stability of the communities. Furthermore, our species-based network showed a greater degree of network specialization (*H*_2_′), indicating a higher level of niche partitioning, and thus less redundancy (Blüthgen, Menzel & Blüthgen, 2006) across taxonomic nodes when compared to the trait-based nodes. While this may also be influenced by some aspects of specialization being missed because other traits that might be important for bee preferences were not included in our analysis, a similar pattern was also observed by Kuppler et al. (2016) when they compared species-averaged and individual based plant-insect networks. It is thereofre possible that additional important information relevant to the resilience of communities (Naeem, 1998; McCann, 2000) may be overlooked if we just examine networks that do not include intraspecific variation in functional traits (Kuppler et al., 2016; Kuppler et al., 2017).

Our trait-based method also revealed a clear hierarchical structure in our mutualistic network that was not so apparent using the traditional species-based approach. We found that nestedness was strongly affected by the level of organisation used to construct the networks. The individual functional size-based network was almost twice as nested as its species-based equivalent (Table 1). This difference in nestedness is important because nestedness reflects functional redundancy, which in turn increases the stability of the system if some of the interactions disappear (Memmott, Waser & Price, 2004; Bascompte & Jordano, 2007; Thébault & Fontaine, 2010).

An important point worth considering is that weighted connectance (*C*_*q*_) and interaction evenness (*IE*) were significantly lower than what would be expected in an assemblage where interactions are randomly assigned among nodes. The notable exception was network specialization (*H*_2_′), which was significantly higher in both networks than for random networks. This result could reflect the fact that we have focused our network approach on a subset (bees) of the whole range of flower visitors present in the community. However, to explore this further it will be necessary to apply our trait-based approach to all flower visitors in multiple communities.

At the node level, the species network showed greater bee specialization (*d*′) than the functional size-based network. A similar pattern has also been observed by Kuppler et al. (2016). In their study, they constructed an individual plant-flower visitor network where plant nodes represented individual plants of a single species and bee nodes represented species of flower visitors. When they compared specialization of individual plants from one species with specialization of plant species within traditional species-averaged plant-flower visitor networks, they found that individual plants were substantially less specialized than plant species. These results therefore indicate that traditional species-averaged networks may be overestimating specialization and that by focusing on individual interactions we may find that mutualistic networks have higher levels of redundancy than previously thought. The taxonomic network in our study also showed a significantly lower value of closeness centrality than the trait-based network, indicating a lower cohesion of the interacting community. Central nodes help to identify keystone species (Martín González, Dalsgaard & Olesen, 2010; Mello et al., 2015), or functional trait groups in the case of trait-based networks. Accordingly, in the species-based network, *B. lucorum* (Apidae) and *C. nigra* (Asteraceae) stood out by their central position among bees and plants, respectively. The trait-based network, however, showed that medium sized bees (3.69–3.83 mm) and plants with NHD ranging from 3.21 to 4.15 mm were key to increase the cohesion of the entire networks. Therefore, this network revealed other central species than those identified in the taxonomic network: *A. mellifera*, *B. lapidarius* and *B. pascuorum* (Apidae) in the case of the bees and *C. nigra*, *L. vulgare* (Asteraceae) in the case of the plants.

When the functional trait-based network was constructed without constraining its size by the number of interacting species, we obtained a much simpler web, in which the network parameters analysed significantly differed from the null models in the same way as the constrained functional trait-based network. The centrality analysis also revealed that bee species composing keystone nodes were those with intermediate intertegular distances (ranging from 3.20 to 5.05). Mirroring this pattern, *C. nigra* and *L. vulgare* flowers showing intermediate nectar holder tubes (3.02 to 4.15 mm) were central for maintaining network cohesion.

## Conclusions

In this study we have provided a new analytical framework for assessing mutualistic networks at the entire community level using relevant functional traits of individuals as the level of organisation. We used of cluster analysis, which allows the creation of unevenly spaced trait classes (therefore avoiding the existence of empty classes). The resultant individual-trait based networks can be analysed in the same way as traditional species-based networks to provide a different perspective of plant-animal interactions. Through this approach, we do not intend to foster the replacement of the classic taxonomic approach, but to open the possibility of having complementary information through different assemblage versions of the same community. Species-based networks allow us to characterize the role of taxonomy in determining the presence and patterning of the interactions, whereas trait-based networks may better capture the importance of functional traits and intraspecific variability in shaping the structure of the interactions (Woodward et al., 2010b).

##  Supplemental Information

10.7717/peerj.5618/supp-1 Supplemental Information 1 Details of the species of plants and bees sampled, and members of the trait nodes

10.7717/peerj.5618/supp-2 Supplemental Information 2 R code for constructing the trait-based networks

10.7717/peerj.5618/supp-3 Data S1 Bee-plant interactions and trait measurements used in our analysis

## Additional Information and Declarations

### Data Availability

The following information was supplied regarding data availability: The raw data and code are provided in the Supplemental Files.
