# Supplementary material for: Zooming into plant-flower visitor networks: an individual trait-based approach
Source: PeerJ. 2018 Sep 17;6:e5618. doi: 10.7717/peerj.5618 (PMC6147118; doi:10.7717/peerj.5618)
Supplement: Supplemental Information 1 [file peerj-06-5618-s001.pdf]

Zooming into plant-flower visitor networks: an individual trait-based approach

Rumeu B, Sheath DJ, Hawes JE and Ings TC*

*Correspondence to:

thomas.ings@anglia.ac.uk

Electronic Supplementary Material

Contents:

Table S1. Plant species list ..………….. 2

Table S2. Bee species list …...………... 3

Table S3. Plant nodes (constrained) 4

Table S4. Bee nodes (constrained) ....……………………………... 5

Table S5. Nestedness (WNODF) ………………………………….. 6

Table S6. Centrality (constrained) …………………………………. 7

Table S7. Plant nodes (unconstrained) 8

Table S8. Bee nodes (unconstrained) ..…………………………….. 8

Table S9. Centrality (unconstrained) ……………………………… 9

Fig. S1. Transect maps ………………..……………………………. 10

Fig. S2. Functional size relationships ....………………………… 11

Fig. S3. Size variation in bee and plant nodes (constrained) ………. 12

Fig. S4. Interaction matrices (species-based and constrained) …….. 13

Fig. S5. Size variation in bee and plant nodes (unconstrained) …… 14

Fig. S6. Interaction matrix (unconstrained) ………………………... 15

Table S1. List of the plant species recorded, sorted from lowest to highest average nectar holder depth. Mean values represent predicted nectar holder depths for flowers recorded during the interactions.

**Species**	**Family**	**Nectar holder depth (mean ± SD [n])**
Heracleum sphondylium (L.)	Apiaceae	1.00* ± 0.00 [11]
Hypochaeris radicata (L.)	Asteraceae	1.00* ± 0.00 [11]
Ranunculus repens (L.)	Ranunculaceae	1.00* ± 0.00 [20]
Taraxacum officinale (L.) Weber ex F.H.Wigg.	Asteraceae	1.00* ± 0.00 [1]
Leucanthemum vulgare (Lam.)	Asteraceae	3.46 ± 0.07 [17]
Centaurea nigra (L.)	Asteraceae	3.70 ± 0.21 [154]
Trifolium repens (L.)	Fabaceae	5.48 ± 0.35 [21]
Lathyrus pratensis (L.)	Fabaceae	7.40† ± 0.00 [5]
Cirsium arvense (L.) Scoop.	Asteraceae	8.45 ± 0.00 [2]
Trifolium pratense (L.)	Fabaceae	10.00† ± 0.00 [30]

* the nectar holder depth of open flowers and those with nectar holder depths too short to effectively measure were assigned a value of 1mm.

† as the correlation between nectar holder depth and flower size was not significant, all flowers were given the mean value of the nectar holder depth of the measured subsample.

Table S2. List of the bee species recorded, sorted from lowest to highest average intertegular distance.

Species	Family	Intertegular distance (mean ± SD (mm) [n])
Lasioglossum pauxillum (Schenck, 1853)	Halictidae	1.37 ± 0.00 [1]
Andrena semilaevis (Pérez, 1903)	Andrenidae	1.73 ± 0.13 [3]
Andrena minutula (Kirby, 1802)	Andrenidae	1.76 ± 0.20 [4]
Lasioglossum malachurum (Kirby, 1802)	Halictidae	1.87 ± 0.20 [12]
Sphecodes ephippius (Linnaeus, 1767)	Halictidae	1.88 ±0.14 [2]
Halictus tumulorum (Linnaeus, 1758)	Halictidae	1.91 ± 0.00 [1]
Lasioglossum puncticolle (Morawitz, 1872)	Halictidae	1.92 ± 0.00 [1]
Nomada flavoguttata (Kirby, 1802)	Apidae	1.97 ± 0.00 [1]
Lasioglossum calceatum (Scopoli, 1763)	Halictidae	2.08 ± 0.16 [11]
Andrena ovatula (Kirby, 1802)	Andrenidae	2.29 ± 0.39 [2]
Andrena chrysosceles (Kirby, 1802)	Andrenidae	2.35 ± 0.14 [9]
Andrena fulvago (Christ, 1791)	Andrenidae	2.41 ± 0.14 [3]
Andrena alfkenella (Perkins, 1914)	Andrenidae	2.54 ± 0.00 [1]
Andrena dorsata (Kirby, 1802)	Andrenidae	2.75 ± 0.24 [2]
Andrena wilkella (Kirby, 1802)	Andrenidae	2.84 ± 0.00 [1]
Colletes daviesanus (Smith, 1846)	Colletidae	2.92 ± 0.00 [1]
Melitta leporina (Panzer, 1799)	Melittidae	3.25 ± 0.00 [1]
Andrena flavipes (Panzer, 1799)	Andrenidae	3.27 ± 0.12 [10]
Andrena nigroaenea (Kirby, 1802)	Andrenidae	3.515±0.00 [1]
Apis mellifera (Linnaeus, 1758)	Apidae	3.59 ± 0.21 [49]
Megachile versicolor (Smith, F., 1844)	Megachilidae	3.63 ± 0.00 [1]
Bombus pascuorum (Scopoli, 1763)	Apidae	4.30 ± 0.69 [19]
Bombus lapidarius (Linnaeus, 1758)	Apidae	4.34 ± 0.41 [84]
Bombus lucorum (Linnaeus, 1761)	Apidae	4.61 ± 0.40 [8]
Bombus vestalis (Geoffroy, 1785)	Apidae	4.77 ± 0.30 [5]
Megachile ligniseca (Kirby, 1802)	Megachilidae	4.85 ± 0.56 [4]
Bombus hortorum (Linnaeus, 1761)	Apidae	4.89 ± 0.87 [5]
Bombus terrestris (Linnaeus, 1758)	Apidae	5.01 ± 0.60 [30]

Table S3. Details on the plant nodes of the *constrained* functional size-based network. Nodes are sorted from lowest to highest ranges of nectar holder depth.

Flower-nodes	No. links	Nectar holder depth (mm)	No. families	Families	No. spp	Species
F01	43	1.00	3	Apiaceae, Asteraceae, Ranunculaceae	4	*Heracleum sphondylium*, *Hypochaeris radicata*, *Ranunculus rapens*, *Taraxacum officinale*
F02	2	3.02 – 3.11	1	Asteraceae	1	*Centaurea nigra*
F03	168	3.21 – 4.15	1	Asteraceae	2	*Centaurea nigra*, *Leucanthemum vulgare*
F04	1	4.58	1	Asteraceae	1	*Centaurea nigra*
F05	13	4.87 – 5.43	1	Fabaceae	1	*Trifolium repens*
F06	7	5.65 – 6.05	1	Fabaceae	1	*Trifolium repens*
F07	1	6.382	1	Fabaceae	1	*Trifolium repens*
F08	5	7.40	1	Fabaceae	1	*Lathyrus pratensis*
F09	2	8.45	1	Asteraceae	1	*Cirsium arvense*
F10	30	10.00	1	Fabaceae	1	*Trifolium pratense*

Table S4. Details on the bee-nodes of the *constrained* functional size-based network. Nodes are sorted from lowest to highest non-overlapping ranges of intertegular distance.

Bee-nodes	No. links	Intertegular distance (mm)	No. families	Families	No. spp	Species
B01	2	1.37 – 1.39	1	Halictidae	2	*Lasioglossum malachurum, L. pauxillum*
B02	2	1.53 – 1.60	1	Andrenidae	2	*Andrena minutula, A. semilaevis*
B03	10	1.70 – 1.81	2	Andrenidae, Halictidae	5	*Andrena minutula, A. semilaevis, Lasioglossum calceatum, L. malachurum, Sphecodes ephippius*
B04	16	1.87 – 2.04	3	Andrenidae, Halictidae, Apidae	9	*Andrena minutula, A. ovatula, A. semilaevis, Halictus tumulorum, Lasioglossum calceatum, L. malachurum, L. puncticolle, Nomada flavoguttata, Sphecodes ephippius*
B05	9	2.08 – 2.27	2	Andrenidae, Halictidae	4	*Andrena chrysosceles, Andrena fulvago, Lasioglossum calceatum, Lasioglossum malachurum*
B06	8	2.36 – 2.44	2	Andrenidae, Halictidae	3	*Andrena chrysosceles, A. fulvago, Lasioglossum calceatum*
B07	5	2.54 – 2.58	1	Andrenidae	3	*Andrena alfkenella, A. chrysosceles, A. dorsata*
B08	3	2.84 – 2.92	2	Andrenidae, Colletidae	3	*Andrena dorsata, A. wilkella, Colletes daviesanus*
B09	2	3.04 – 3.10	1	Andrenidae	1	*Andrena flavipes*
B10	16	3.20 – 3.35	3	Andrenidae, Apidae, Melittidae	4	*Andrena flavipes, Apis mellifera, Bombus pascuorum, Melitta leporina*
B11	9	3.38 – 3.47	2	Andrenidae, Apidae	3	*Andrena flavipes, Apis mellifera, Bombus lapidarius*
B12	27	3.49 – 3.67	3	Andrenidae, Apidae, Megachilidae	5	*Andrena nigroaenea, Apis mellifera, Bombus lapidarius, B. pascuorum, Megachile versicolor*
B13	19	3.69 – 3.83	1	Apidae	3	*Apis mellifera, Bombus lapidarius, B. pascuorum*
B14	15	3.88 – 4.03	1	Apidae	4	*Apis mellifera, Bombus lapidarius, B. pascuorum, B. terrestris*
B15	10	4.06 – 4.12	1	Apidae	1	*Bombus lapidarius*
B16	38	4.16 – 4.39	2	Apidae, Megachilidae	7	*Apis mellifera, Bombus hortorum, B. lapidarius, B. lucorum, B. pascuorum, B. terrestris, Megachile ligniseca*
B17	18	4.42 – 4.57	1	Apidae	6	*Bombus hortorum, B. lapidarius, B. lucorum, B. pascuorum, B. terrestris, B. vestalis*

Table S4 continued

Bee-nodes	No. links	Intertegular distance (mm)	No. families	Families	No. spp	Species
B18	16	4.59 – 4.74	1	Apidae	5	*Bombus lapidarius, B. lucorum, B. pascuorum, B. terrestris, B. vestalis*
B19	14	4.77 – 4.86	2	Apidae, Megachilidae	6	*Bombus lapidarius, B. lucorum, B. pascuorum, B. terrestris, B. vestalis, Megachile ligniseca*
B20	9	4.88 – 5.05	1	Apidae	3	*Bombus lapidarius, B. lucorum, B. terrestris*
B21	10	5.13 – 5.31	1	Apidae	5	*Bombus hortorum, B. lapidarius, B. lucorum, B. terrestris, B. vestalis*
B22	4	5.42 – 5.50	1	Apidae	1	*Bombus terrestris*
B23	5	5.58 – 5.68	2	Apidae, Megachilidae	3	*Bombus pascuorum, B. terrestris, Megachile ligniseca*
B24	1	5.76	1	Apidae	1	*Bombus terrestris*
B25	1	5.90	1	Apidae	1	*Bombus terrestris*
B26	1	6.04	1	Apidae	1	*Bombus pascuorum*
B27	1	6.23	1	Apidae	1	*Bombus hortorum*
B28	1	6.42	1	Apidae	1	*Bombus terrestris*

Table S5. Nestedness significance levels for the species-based network, its equivalent trait-based network, and the unconstrained trait-based network. Mean WNODF obtained from 1000 randomizations. CRT: Conserve Row Totals, CCT: Conserve Column Totals, and RCTA: Row Column Total Average (see Beckett, et al. 2014).

	**Species-based network**			**Constrained trait-based network**			**Unconstrained trait-based network**		
Null model	WNODF observed	Mean WNODF	*P*	WNODF observed	Mean WNODF	*P*	WNODF observed	Mean WNODF	*P*
CRT	17.382	19.350	0.781	33.324	29.008	0.109	51.041	53.283	0.659
CCT	17.382	25.223	1	33.324	35.693	0.904	51.041	39.078	0.093
RCTA	17.382	24.264	1	33.324	33.553	0.554	51.041	49.483	0.462

Table S6. Centrality metrics calculated for nodes of the species-based network and its equivalent constrained functional size-based network. Most central species shown in bold.

	Species-based network				Constrained trait-based network			
**Level**	Species code	Normalized degre	Closeness	Betweenness	Node code	Normalized degree	Closeness	Betweenness
Plant	*Heracleum sphondylium*	0.14	0.30	0.06	F01	0.50	0.50	0.26
	Hypochaeris radicata	0.25	0.40	0.14	F02	0.07	0.34	0.00
	Ranunculus repen	0.43	0.47	0.31	**F03**	**0.82**	**0.64**	**0.52**
	Taraxacum officinale	0.04	0.32	0.00	F04	0.04	0.26	0.00
	Leucanthemum vulgare	0.29	0.41	0.16	F05	0.36	0.44	0.08
	Centaurea nigra	**0.46**	**0.46**	**0.31**	F06	0.18	0.37	0.01
	Trifolium repens	0.36	0.43	0.24	F07	0.04	0.33	0.00
	Lathyrus pratensis	0.11	0.34	0.00	F08	0.14	0.36	0.01
	Cirsium arvense	0.04	0.32	0.00	F09	0.07	0.34	0.00
	Trifolium pratense	0.14	0.34	0.01	F10	0.43	0.43	0.16
Bee	*Lasioglossum pauxillum*	0.10	0.32	0.00	B01	0.20	0.44	0.01
	Andrena semilaevis	0.20	0.34	0.02	B02	0.10	0.34	0.00
	Andrena minutula	0.30	0.36	0.04	B03	0.20	0.44	0.01
	Lasioglossum malachurum	0.50	0.46	0.13	B04	0.30	0.45	0.01
	Sphecodes ephippius	0.20	0.36	0.01	B05	0.30	0.45	0.01
	Halictus tumulorum	0.10	0.29	0.00	B06	0.20	0.44	0.01
	Lasioglossum puncticolle	0.10	0.29	0.00	B07	0.30	0.38	0.01
	Nomada flavoguttata	0.10	0.32	0.00	B08	0.30	0.42	0.01
	Lasioglossum calceatum	0.40	0.43	0.07	B09	0.20	0.34	0.05
	Andrena ovatula	0.10	0.30	0.00	B10	0.20	0.41	0.00
	Andrena chrysosceles	0.30	0.37	0.05	B11	0.20	0.41	0.00
	Andrena fulvago	0.20	0.34	0.01	B12	0.30	0.44	0.01
	Andrena alfkenella	0.10	0.23	0.00	**B13**	**0.60**	**0.49**	**0.12**
	Andrena dorsata	0.10	0.30	0.00	B14	0.30	0.47	0.02
	Andrena wilkella	0.10	0.30	0.00	B15	0.40	0.46	0.05
	Colletes daviesanus	0.10	0.29	0.00	B16	0.50	0.50	0.05
	Melitta leporina	0.10	0.30	0.00	B17	0.60	0.51	0.07
	Andrena flavipes	0.30	0.42	0.03	B18	0.30	0.43	0.01
	Andrena nigroaenea	0.10	0.29	0.00	B19	0.50	0.50	0.05
	Apis mellifera	0.20	0.36	0.01	B20	0.30	0.43	0.01
	Megachile versicolor	0.10	0.32	0.00	B21	0.20	0.42	0.01
	Bombus pascuorum	0.40	0.36	0.03	B22	0.20	0.42	0.01
	Bombus lapidarius	0.50	0.46	0.13	B23	0.20	0.42	0.01
	Bombus lucorum	**0.60**	**0.47**	**0.14**	B24	0.10	0.30	0.00
	Bombus vestalis	0.20	0.36	0.01	B25	0.10	0.39	0.00
	Megachile ligniseca	0.10	0.32	0.00	B26	0.10	0.30	0.00
	Bombus hortorum	0.20	0.32	0.00	B27	0.10	0.39	0.00
	Bombus terrestris	0.50	0.44	0.07	B28	0.10	0.39	0.00

Table S7. Details on the plant nodes of the *unconstrained* functional size-based network where the number of nodes was obtained independently of the number of interacting species. Nodes are sorted from lowest to highest ranges of nectar holder depth.

Flower-nodes	No. links	Nectar holder depth (mm)	No. families	Families	No. spp	Species
F01	43	1.00	3	Apiaceae, Asteraceae, Ranunculaceae	4	*Heracleum sphondylium, Hypochaeris radicata, Ranunculus repens, Taraxacum officinale*
F02	170	3.02 – 4.15	1	Asteraceae	2	*Centaurea nigra, Leucanthemum vulgare*
F03	22	4.58 – 6.38	2	Asteraceae, Fabaceae	2	*Centaurea nigra, Trifolium repens*
F04	7	7.40 – 8.45	2	Asteraceae, Fabaceae	2	*Cirsium arvense, Lathyrus pratensis*
F05	30	10.00	1	Fabaceae	1	*Trifolium pratense*

Table S8. Details on the bee-nodes of the *unconstrained* functional size-based network where the number of nodes was obtained independently of the number of interacting species. Nodes are sorted from lowest to highest non-overlapping ranges of intertegular distance.

Bee-nodes	No. links	Intertegular distance (mm)	No. fam.	Families	No. spp	Species
B01	57	1.37 – 3.10	3	Andrenidae, Halictidae, Apidae	17	*Andrena alfkenella, A. chrysosceles, A. dorsata, A. flavipes, A. fulvago, A. minutula, A. ovatula, A. semilaevis, A. wilkella, Colletes daviesanus, Halictus tumulorum, Lasioglossum calceatum, L. malachurum, L. pauxillum, L. puncticolle, Nomada flavoguttata, Sphecodes ephippius*
B02	71	3.20 – 3.83	4	Andrenidae, Apidae, Megachilidae, Melittidae	7	*Andrena flavipes, A. nigroaenea, Apis mellifera, Bombus lapidarius, B. pascuorum, Megachile versicolor, Melitta leporina*
B03	120	3.88 – 5.05	2	Apidae, Megachilidae	8	*Apis mellifera, Bombus hortorum, Bombus lapidarius, Bombus lucorum, Bombus pascuorum, Bombus terrestris, Bombus vestalis, Megachile ligniseca*
B04	24	5.13 – 6.42	2	Apidae, Megachilidae	7	*Bombus hortorum, B. lapidarius, B. lucorum, B. pascuorum, B. terrestris, B. vestalis, Megachile ligniseca*

Table S9. Centrality metrics calculated for nodes of the unconstrained trait-based network in which the number of nodes was obtained independently of the number of interacting species. Most central nodes shown in bold.

	Unconstrained trait-based network			
**Level**	Node code	Normalized degree	Closeness	Betweenness
Plant	F01	0.75	0.57	0.05
	**F02**	**1.00**	**0.67**	**0.19**
	F03	0.75	0.57	0.05
	F04	0.50	0.50	0.01
	F05	0.75	0.57	0.09
Bee	B01	0.60	0.53	0.05
	**B02**	**1.00**	**0.73**	**0.23**
	**B03**	**1.00**	**0.73**	**0.23**
	B04	0.40	0.47	0.02

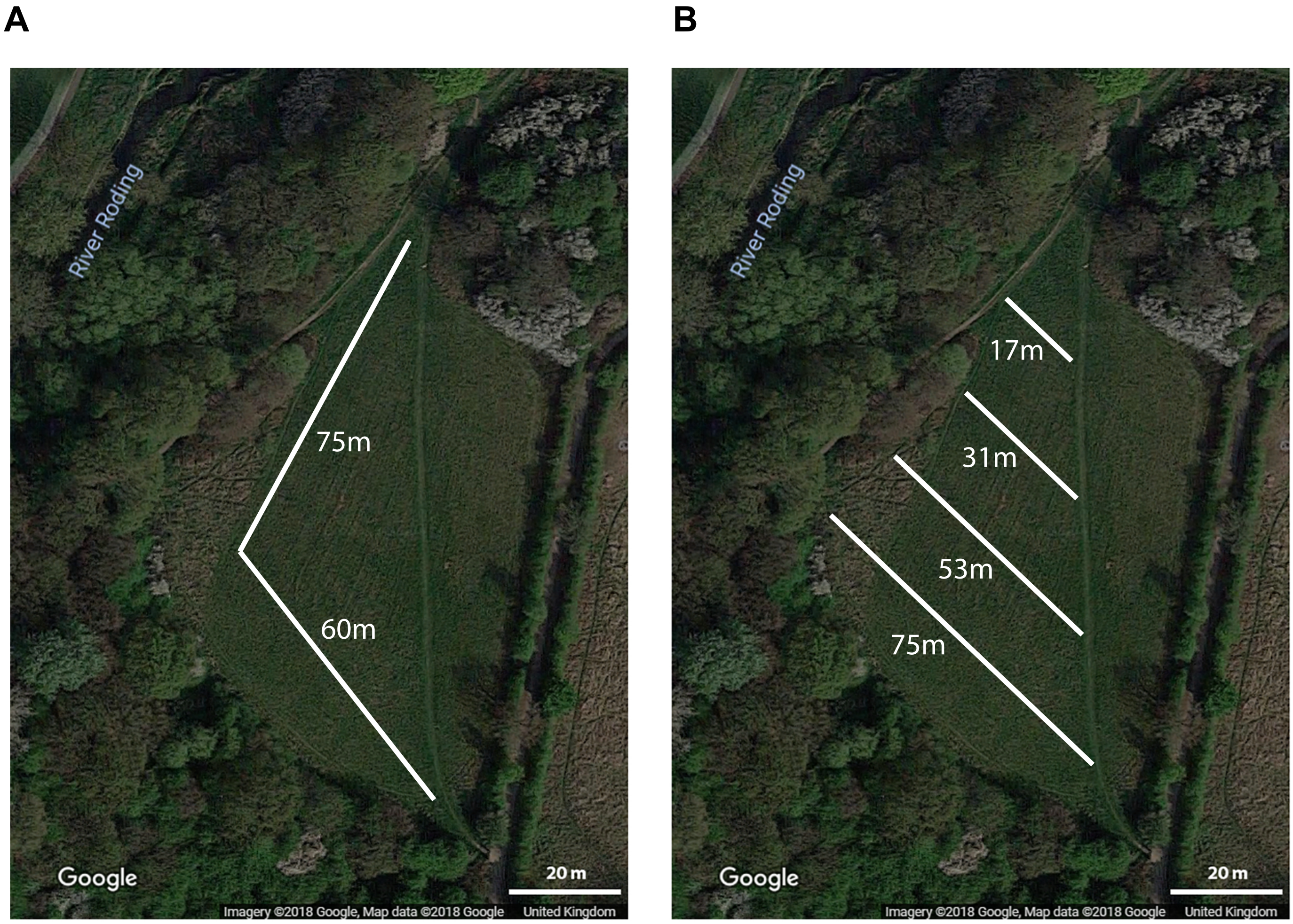

Fig. S1. Aerial photograph (Map data © 2018 Google) of the study site at the Roding Valley Nature Reserve (Essex, UK [51°38'04.1"N 0°04'12.6"E]) showing the location of the transects used to monitor interactions between bees and flowers in (A) 2011 and (B) 2012. The transects were positioned to maximise the floral diversity and be representative of the whole meadow. North is to the top of the images.

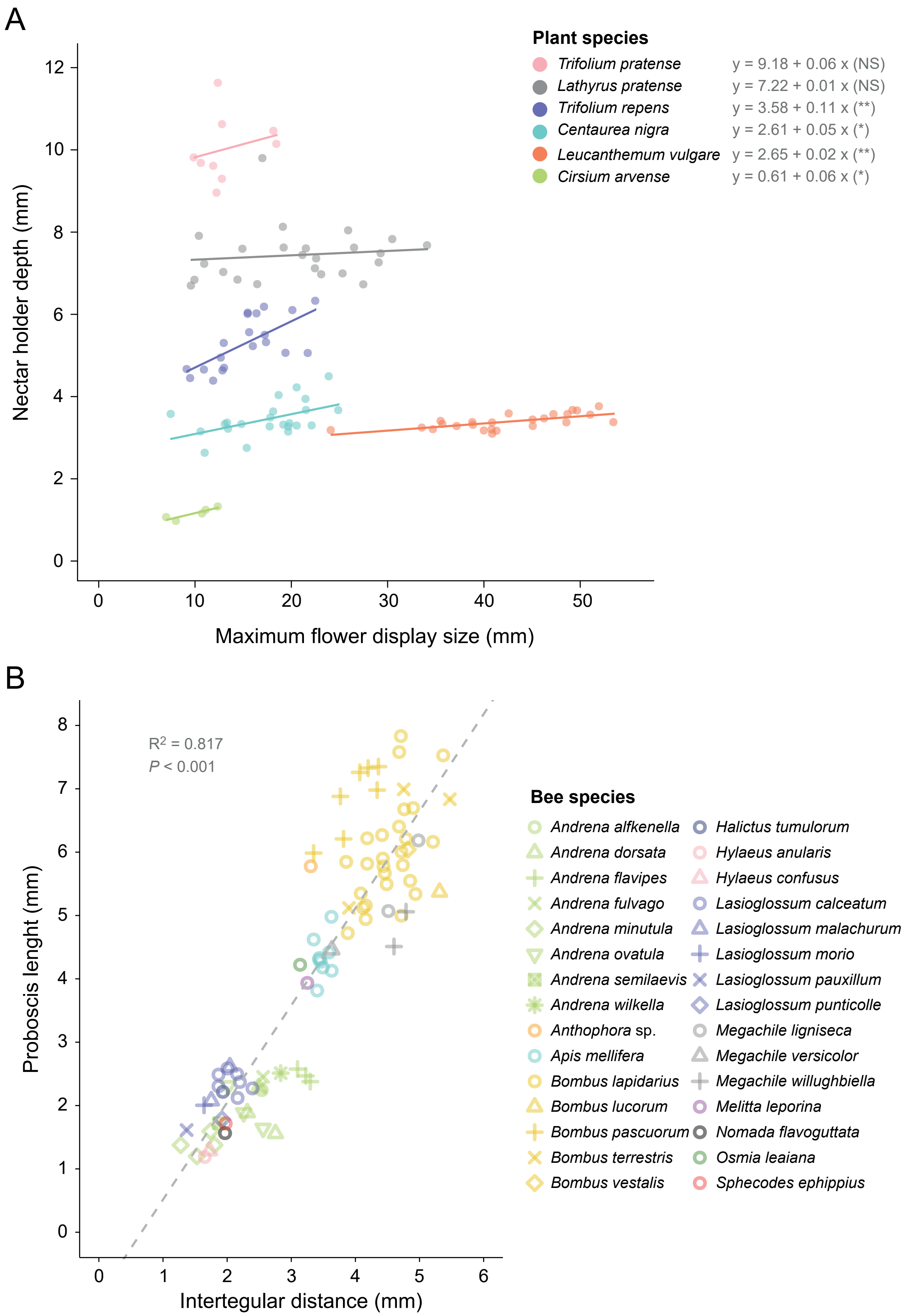

Fig. S2. (A) Relationship between maximum floral display size and nectar holder depth in six out of the ten plant species included in the study. The significance of the slopes is shown after the equations for each line, * for P<0.05) and ** for P<0.01, NS for P > 0.05. (B) Relationship between intertegular distance and proboscis length for 30 species of bee, including 27/28 species focused on this study. Colours represent genus. For both panels, each point represents an individual flower or bee, respectively.

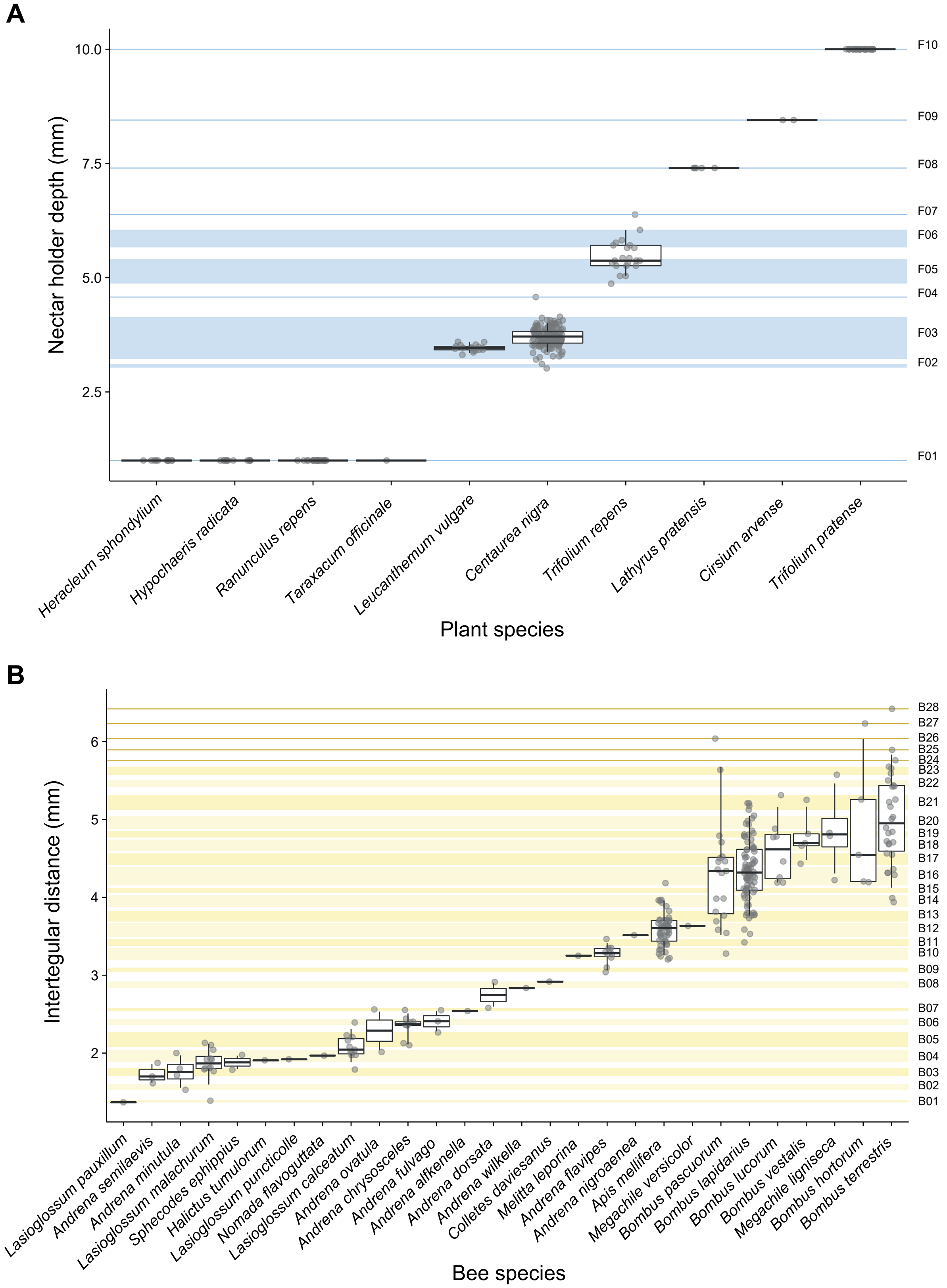

Fig. S3. Boxplot (median, quartiles and 5th/95th percentiles) showing the size variation in A) the nectar holder depth predicted for the flowers (grey dots) of the ten plant species included in the study, and B) the intertegular distance (a proxy of the proboscis length) for the 28 bee species included in the study. Blue and yellow bands group the flowers and bees according to the agglomerative hierarchical cluster analysis in which the final number of clusters matches the number of interacting plant species, i.e. the constrained trait-based network.

A

Intertegular distance (– → +)

Nectar holder depth (– ↓ +)

	Lasioglossum pauxillum	Andrena semilaevis	Andrena minutula	Lasioglossum malachurum	Sphecodes ephippius	Halictus tumulorum	Lasioglossum puncticolle	Nomada flavoguttata	Lasioglossum calceatum	Andrena ovatula	Andrena chrysosceles	Andrena fulvago	Andrena alfkenella	Andrena dorsata	Andrena wilkella	Colletes daviesanus	Melitta leporina	Andrena flavipes	Andrena nigroaenea	Apis mellifera	Megachile versicolor	Bombus pascuorum	Bombus lapidarius	Bombus lucorum	Bombus vestalis	Megachile ligniseca	Bombus hortorum	Bombus terrestris
Heracleum sphondylium		1	2								7		1															
Hypochaeris radicata			1	1		1	1		1			2											4					
Ranunculus repens		2	1	6	1			1	3		1	1						1					1	1				1
Taraxacum officinale				1																								
Leucanthemum vulgare					1				2		1					1			1	8				1	2			
Centaurea nigra	1			3					5									8		41	1	8	68	2	3	4	1	9
Trifolium repens				1						2				2	1		1	1				2	9	1				1
Lathyrus pratensis																						2		2				1
Cirsium arvense																							2					
Trifolium pratense																						7		1			4	18

B

Intertegular distance (– → +)

Nectar holder depth (– ↓ +)

	B01	B02	B03	B04	B05	B06	B07	B08	B09	B10	B11	B12	B13	B14	B15	B16	B17	B18	B19	B20	B21	B22	B23	B24	B25	B26	B27	B28
F01	1	2	7	10	6	6	3		1				1	1	1	2	1		1									
F02													1		1													
F03	1		3	5	2	2		1		15	8	25	12	12	7	25	10	12	9	6	8	1	1		1		1	1
F04									1																			
F05				1			1	1		1	1	1	3			2	1		1									
F06					1		1	1								3	1											
F07													1															
F08																	1	1	1	2								
F09													1		1													
F10												1		2		6	4	3	2	1	2	3	4	1		1		

Fig. S4. Plant-bee interaction matrices for the species-based network (A) and the constrained functional trait-based network (B). Species and clusters are sorted from lowest to highest size values (in the case of interaction matrix A mean values have been considered). See Tables S1-S4 for details in trait variation per species or flower- and bee-nodes. Filled cells indicate presence of interactions. The intensity of the filled cells denotes a higher frequency of interactions. Numbers indicate the frequency of interactions.

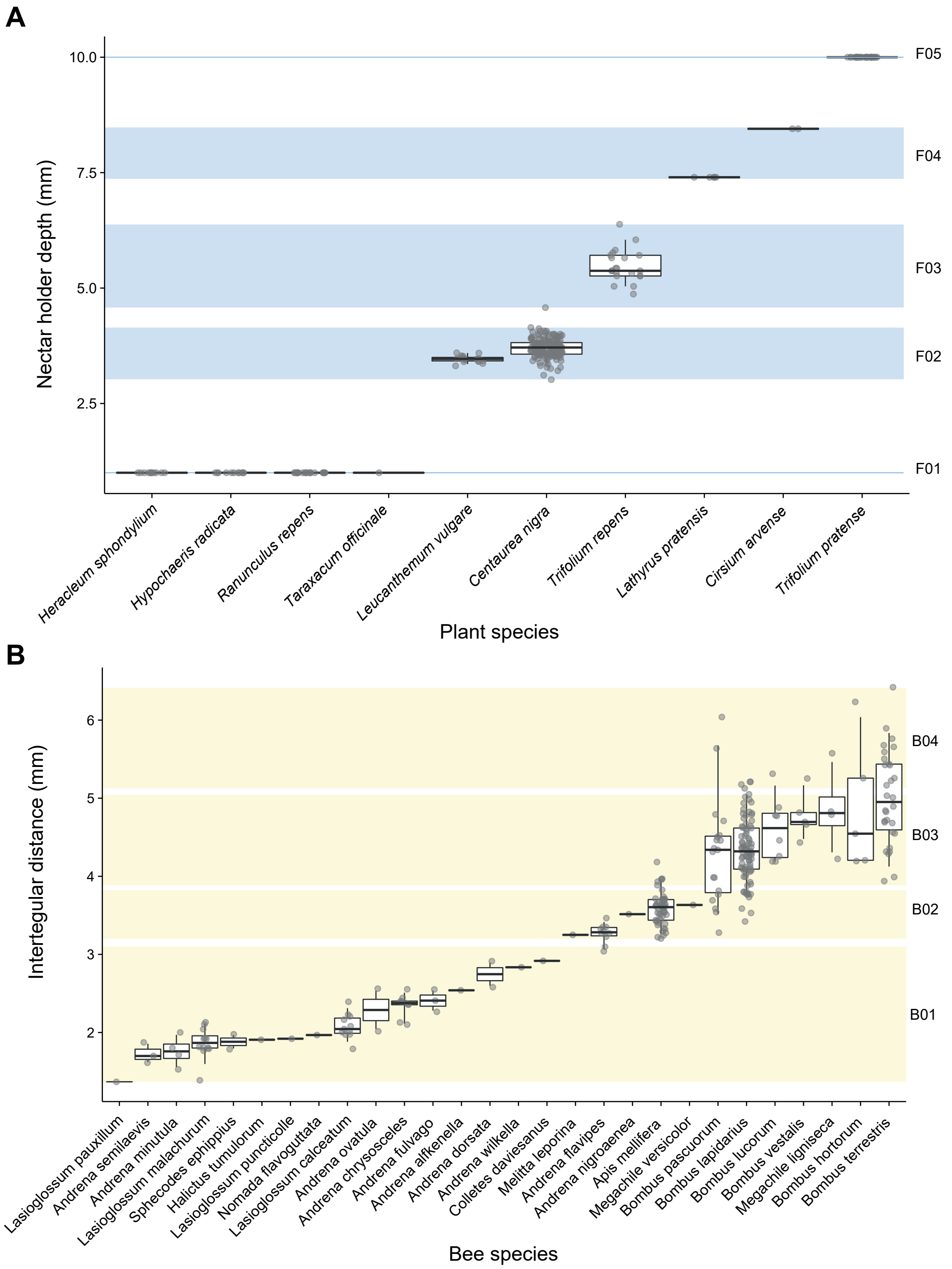

Fig. S5. Boxplot (median, quartiles and 5th/95th percentiles) showing the size variation in A) the nectar holder depth predicted for the flowers (grey dots) of the ten plant species included in the study, and B) the intertegular distance (a proxy of the proboscis length) for the 28 bee species included in the study. Blue and yellow bands group the flowers and bees according to the agglomerative hierarchical cluster analysis in which the final number of clusters have been optimized by the analysis - i.e. the unconstrained trait-based network.

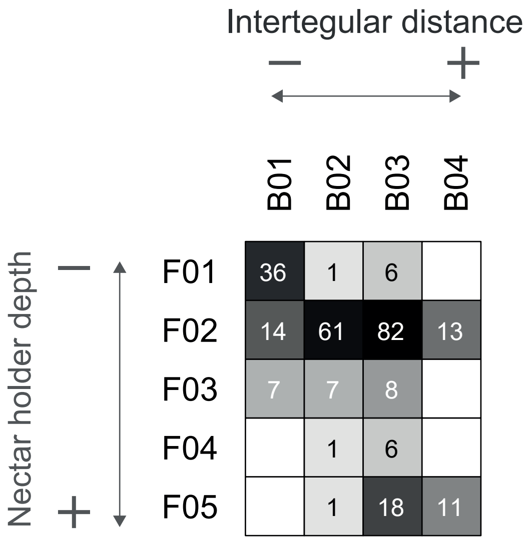

Fig. S6. Plant-bee interaction matrix for the unconstrained trait-based network. Plant- and Bee- clusters are sorted from lowest to highest size values. See Tables S7-8 for details in trait variation per plant- and bee-node. Filled cells indicate presence of interactions. The intensity of the filled cells denotes a higher frequency of interactions. Numbers indicate the frequency of interactions.

References

Beckett, S. J., et al. 2014. FALCON: a software package for analysis of nestedness in bipartite networks. - F1000Research 3: 185.
